# Variable sensitivity to complement-dependent cytotoxicity in murine models of neuromyelitis optica

**DOI:** 10.1186/s12974-016-0767-4

**Published:** 2016-12-01

**Authors:** Yiting Liu, Danielle E. Harlow, Katherine S. Given, Gregory P. Owens, Wendy B. Macklin, Jeffrey L. Bennett

**Affiliations:** 1 Department of Neurology, University of Colorado, School of Medicine, 12700 E. 19th Ave, Aurora, CO USA; 2 Department of Cell & Developmental Biology, University of Colorado, School of Medicine, 12700 E. 19th Ave, Aurora, CO USA; 3 Department of Ophthalmology, University of Colorado, School of Medicine, 12700 E. 19th Ave, Aurora, CO USA; 4 Program in Neuroscience, University of Colorado, School of Medicine, 12700 E. 19th Ave, Aurora, CO USA

**Keywords:** NMO, Complement, Cytotoxicity, Mouse model, Neuroglial networks

## Abstract

**Background:**

Studies of neuromyelitis optica (NMO), an autoimmune disease of the central nervous system (CNS), have demonstrated that autoantibodies against the water channel aquaporin-4 (AQP4) induce astrocyte damage through complement-dependent cytotoxicity (CDC). In developing experimental models of NMO using cells, tissues or animals from mice, co-administration of AQP4-IgG and normal human serum, which serves as the source of human complement (HC), is required. The sensitivity of mouse CNS cells to HC and CDC in these models is not known.

**Methods:**

We used HC and recombinant monoclonal antibodies (rAbs) against AQP4 to investigate CDC on mouse neurons, astrocytes, differentiated oligodendrocytes (OLs), and oligodendrocyte progenitors (OPCs) in the context of purified monocultures, neuroglial mixed cultures, and organotypic cerebellar slices.

**Results:**

We found that murine neurons, OLs, and OPCs were sensitive to HC in monocultures. In mixed murine neuroglial cultures, HC-mediated toxicity to neurons and OLs was reduced; however, astrocyte damage induced by an AQP-specific rAb #53 and HC increased neuronal and oligodendroglial loss. OPCs were resistant to HC toxicity in neuroglial mixed cultures. In mouse cerebellar slices, damage to neurons and OLs following rAb #53-mediated CDC was further reduced, but in contrast to neuroglial mixed cultures, astrocyte damage sensitized OPCs to complement damage. Finally, we established that some injury to neurons, OLs, and OPCs in cell and slice cultures resulted from the activation of HC by anti-tissue antibodies to mouse cells.

**Conclusions:**

Murine neurons and oligodendroglia demonstrate variable sensitivity to activated complement based on their differentiation and culture conditions. In organotypic cultures, the protection of neurons, OLs, and OPCs against CDC is eliminated by targeted astrocyte destruction. The activation of human complement proteins on mouse CNS cells necessitates caution when interpreting the results of mouse experimental models of NMO using HC.

**Electronic supplementary material:**

The online version of this article (doi:10.1186/s12974-016-0767-4) contains supplementary material, which is available to authorized users.

## Background

Neuromyelitis optica (NMO) is a severe autoimmune disease of the central nervous system (CNS) associated with predilection for the spinal cord and optic nerve. Histopathology and animal models demonstrate that complement-dependent cytotoxicity (CDC) plays a key role in the initiation of the NMO pathology [1].

The complement system consists of over 30 components that are normally found inactive in serum and is an essential immune regulator of host defense to infection, cell integrity, and tissue homeostasis. The complement cascade can be activated by antigen-antibody complexes (classic pathway) or the antibody-independent lectin (lectin pathway). Additionally, the alternative pathway serves to amplify the classic and lectin pathways. All three pathways converge to trigger activation of terminal complement components resulting in cell lysis [2]. In the CNS, the complement cascade regulates synaptic refinement and neuronal survival during development [3, 4] and plays a role in maintaining brain homeostasis in adulthood [5]. There is mounting evidence that complement synthesis and activation are increased in neurodegenerative and neuroinflammatory environments [6].

NMO lesions show perivascular deposition of immunoglobulin and activated complement [7, 8], and activated complement proteins are elevated in the serum and cerebrospinal fluid (CSF) of NMO patients [9, 10]. Serum autoantibodies (NMO-IgG) against the water channel aquaporin-4 (AQP4) are found in most NMO patients [11]. In the CSF of NMO-IgG positive patients, there exists a dynamic and enriched population of expanded plasmablast clones producing AQP4-specific antibodies [12]. Both serum NMO-IgG and CSF-derived AQP4-specific monoclonal recombinant antibodies recapitulate NMO pathology when microinjected into the CNS, added to ex vivo spinal cord slices in the presence of human complement proteins [13], introduced into rodent models of CNS inflammation [12, 14], or injected into mice pretreated with Freund’s complete adjuvant [15]. Astrocytes are selectively targeted in NMO, as evidenced by the extensive loss of immunoreactivity for the astrocytic proteins AQP4 and glial fibrillary acidic protein (GFAP) [7]. Moreover, inhibition of the classic complement pathway blocks neuropathology in both in vitro and in vivo models of NMO [16].

In developing experimental mouse models of NMO in vivo, ex vivo, or in vitro, co-administration of AQP4-IgG and normal human serum as the source of human complement (HC) is required to produce NMO pathological lesions [17]. The weak activity of intrinsic mouse complement [18] and the presence of complement inhibitor(s) in the mouse serum [19] may combine to prevent rigorous complement activation in the absence of added HC in NMO models. Additional effects of HC on other CNS cells have not been characterized in NMO animal models.

In this study, we investigated the toxic effects of HC on murine neurons, astrocytes, differentiated oligodendrocytes (OLs), and oligodendrocyte progenitors (OPCs) in the presence or absence of AQP4-IgG in several in vitro and ex vivo CNS culture systems (monocultures, neuroglial mixed cultures, and organotypic cerebellar slice cultures). Our results reveal that neurons, OLs, and OPCs derived from mice display differential sensitivity to HC depending on the complexity of the neuroglial environment, and that AQP4-IgG-targeted astrocyte destruction results in secondary oligodendrocyte and neuronal loss.

## Methods

### Recombinant antibodies

The recombinant monoclonal human anti-AQP4 antibody #53 (rAb #53) was constructed from a clonally expanded plasmablast recovered from the cerebrospinal fluid (CSF) of a seropositive NMO patient. The control human antibody was generated from a CSF plasmablast sorted from a chronic meningitis patient. Recombinant antibodies were produced using a dual vector transient transfection system and purified with protein A-sepharose (Sigma-Aldrich, St. Louis, MO) as previously described [12].

### Biochemical assays and reagents

Lactate dehydrogenase (LDH) cytotoxicity assays were performed on cell culture supernatants using the LDH cytotoxicity detection kit (TakaRa Bio, Shiga, Japan). Except for IncuCyte live imaging (see below), propidium iodide (PI) (Sigma) was used at 5 μg/ml in the culture medium to label dead cells in cell and organotypic slice cultures. Normal, C1q-depleted, C3-depleted, C4-depleted, C5-depleted, and C9-depleted human serum (Complement Technology, Tyler, TX) were used as the source of human complement. Pooled and individual normal human (Sigma and Innovative Research), rat (Complement Technology), and mouse (Complement Technology and Innovative Research) sera were used as sources of complement for comparative complement experiments. Serum proteins were obtained by passing the serum through Amicon Ultra 3KD centrifugal filters (Millipore, Billerica, MA). Protein A/G plus agarose (Thermo Fisher Scientific, Waltham, MA) was incubated with human serum overnight at 4 °C, then centrifuged and discarded to deplete IgGs in the serum. Heat-inactivated serum was obtained by incubating normal serum at 58 °C for 30 min.

### Primary cell cultures

Primary astrocyte, differentiated oligodendrocyte (OL), oligodendrocyte progenitor (OPC), and neuronal monocultures, and neuroglia mixed cultures were prepared as described from mice (CD-1 or PLP-eGFP [20]) or rats (Sprague Dawley) (Harlan, Indianapolis, IN) with some modifications [21–24]. Mixed glial cultures were prepared from P0-1 dissociated cortices and plated in poly-D-lysine-coated 24-well plates at a density of one brain per plate. Plates were used for experiments when astrocyte layer was confluent and oligodendrocyte cell bodies were apparent (day 5–8 in culture). Purified oligodendrocyte monocultures were prepared by shaking flasks of mixed glial cultures overnight at 200 rpm to detach the OPCs. OPCs were plated on poly-D-lysine/laminin coated dishes and maintained in media containing 10 ng/ml PDGF (platelet-derived growth factor)/FGF (fibroblast growth factor) (Peprotech, Rocky Hill, NJ) for 48–72 h prior to experiments. To differentiate OPCs into OLs, PDFG/FGF was withdrawn after 48 h and media supplemented with 40 ng/ml T3 (Sigma). OLs were used for experiments 24–72 h after exposure to T3. For neuroglial mixed cultures, P0-1 cortices were dissected, dissociated in 0.25% trypsin, and cells were plated on poly-D-lysine/Laminin coated coverslips (BD Biosciences, Franklin Lakes, NJ) in Neurobasal medium containing B27 supplement, 0.05 mM L-glutamine, penicillin-streptomycin (all from Life Technologies) and 5% heat-inactivated FBS (Sigma). Neuronal monocultures were prepared from E15-17 mouse cortex and cultured in Neurobasal medium in the presence of B27 supplement, 0.05 mM L-glutamine and penicillin-streptomycin. On day 2–5 in culture, 2 μM ara-C (Sigma) was added to inhibit the growth of glial cells. Human cortical neuron cultures (iCell Neurons) were cultured in iCell proprietary maintenance medium purchased from Cellular Dynamics (Madison, WI). Cell cultures were maintained at 37 °C in 5 or 8.5% CO2 atmospheric conditions.

### Cerebellar slice culture

Sagittal slices (300 μm) were prepared from the cerebella of C57Bl6 or PLP-eGFP mice at P10 and cultured on MilliCell 0.4 μm membrane inserts (Millipore) for 10–14 days in media containing 25% inactivated horse serum [25]. Prior to treatment, slices were switched to a serum-free Neurobasal medium supplemented with B27, 2 mM GlutaMAX, and 28 mM D-glucose (Sigma).

### Immunostaining

#### Differential labeling of cell surface and internalized proteins in cell culture

Cell cultures were washed with fresh culture medium, then incubated with 20 μg/ml recombinant antibody at 4 or 37 °C for 1 h. Cells were then washed and fixed in 4% paraformaldehyde (Thermo Scientific) in phosphate-buffered saline (PBS) and rinsed and blocked with 3% normal goat serum (NGS) and 2% bovine serum albumin (BSA) in PBS without Triton X-100 (all from Sigma). Cells were then probed with Alexa-fluor 488 goat anti-human IgG secondary antibodies (1:500). Subsequently, cells were blocked overnight with unlabeled goat anti-human IgG at 0.25 mg/ml at room temperature and then washed, postfixed, and permeabilized with blocking buffer containing 0.1% Triton X-100. Alexa-fluor 594 goat anti-human IgG (1:500) was used to probe for intracellular recombinant antibody. After extensive washing, slides were mounted with ProLong Gold anti-fade reagent with DAPI (Life Technologies).

#### Immunostaining of fixed cells and brain sclices

Cell cultures or brain slices were fixed in 4% paraformaldehyde in PBS, rinsed, permeabilized in 0.1% (for cells) or 3% (for slices) Triton X-100 in PBS, and then blocked with 5% normal goat serum (NGS) or normal donkey serum (NDS) in PBS containing 0.1% (for cells) or 0.3% (for slices) Triton X-100 for 1 h. Samples were then incubated with primary antibodies in blocking buffer, washed, and then probed with secondary antibodies (Alexa Fluor secondary antibodies, all from Life Technologies). After washing, the coverslips were mounted with ProLong Gold anti-fade reagent (Life Technologies). The brain slices were mounted with Fluoromount-G (Southern Biotech, Birmingham, AL), placed under the glass covers, and sealed. Primary antibodies used in the immunostaining were mouse anti-NeuN (Millipore), mouse anti-β-tubulin (Sigma), rabbit anti-GFAP (DAKO, Carpinteria, CA), mouse anti-Olig1(Millipore), mouse anti-O4 IgM (R&D Systems, Minneapolis, MN ), rabbit anti-LAMP1(Abcam, Cambridge, UK), rabbit anti-Calbindin (Millipore), mouse anti-C3d (a gift from Dr. Joshua Thurman, University of Colorado), rabbit anti-MAC complex (Abcam), and rabbit anti-NG2 and rabbit anti-Olig2 (both are gifts from Dr. Charles Stiles, Harvard University).

### Microscopy

Fluorescence images were acquired by Zeiss fluorescence microscope with Axiovision software (Zeiss, Jena, Germany). Confocal images were acquired by Leica SP5 laser scanning microscope (Leica Microsystems GmbH, Wetzlar, Germany) and Zeiss LSM 780 microscope or Olympus FV-1000 confocal microscope (Olympus, Center Valley, PA).

### IncuCyte live cell imaging

Live cell imaging was performed using IncuCyte Zoom from Essen BioScience (Ann Arbor, MI). Cells were grown and scanned on 24-well plates in the cell culture incubator. Each well was scanned with a 10× objective lens in nine randomly selected positions at 15 min intervals with high definition phase contrast and epifluorescence microscopy using the following filter sets: 460/524 nm (green fluorescence, to detect enhanced green fluorescent protein, eGFP) and 585/635 nm (red fluorescence, to detect the DRAQ7 dead cell nuclei; Cell Signaling Technology, Danvers, MA). Image processing and cell counting were performed using IncuCyte software. Oligodendrocyte lineage cells in the neuroglial mixed cultures were labeled with eGFP in PLP-eGFP mice [20]. In cell culture, OLs and OPCs were defined by their distinct morphologies. In the neuroglia mixed cultures, the majority of oligodendrocytes were bipolar and tripolar OPCs, while a minority of the cells displayed a highly complex network of processes and O4 antigen consistent with differentiating OLs [26]. Neurons and astrocytes were identified by their distinct morphologies. DRAQ7+ cells were classified as dead neurons or astrocytes based on the different signal intensity and area. Dead oligodendrocytes were identified as DRAQ7+ cells with reduced eGFP signal. The identification of different cell types in mixed cultures was confirmed by immunostaining with NeuN (neurons), AQP4 and GFAP (astrocytes), Olig1 (a general oligodendrocyte lineage marker), and O4 (differentiating OLs) (Additional file 1: Figure S1). To assess the total number of the cells in the cultures, cells were stained with Cell Trace Calcein red-orange (Life Technologies) and then scanned and counted using IncuCyte software.

### Quantification and statistical analysis

Cell counts in IncuCyte images were quantified by IncuCyte software. In each well of the 24-well plate, signals were measured in nine scanned positions and summated. PLP-eGFP positive cell bodies in the brain slices were imaged using a Zeiss fluorescence microscope with 20× objective. Images were quantified with ImageJ (National Institutes of Health open source). For each slice, 2–3 images were taken, quantified, and averaged. Slices from three independent experiments were analyzed. Statistical analyses were performed by unpaired Student’s *t* test for single comparisons or by two-way ANOVA for grouped comparisons using GraphPad Prism software. Data are expressed as means ± SD of independent experiments (*n* ≥ 3). Significance is reported for *p* < 0.05.

## Results

### Human serum as the source of human complement is cytotoxic to mouse neurons and oligodendrocytes, but not astrocytes in monocultures

**Fig. 1:**
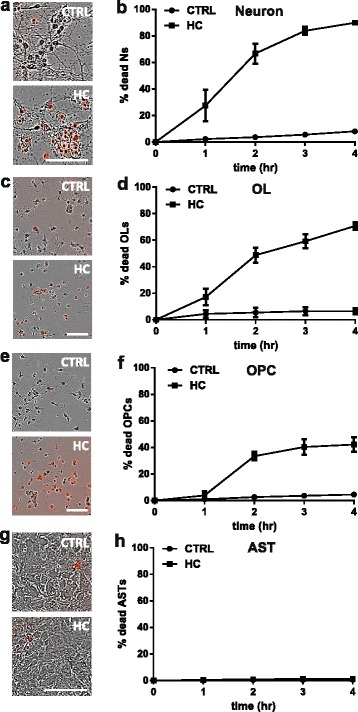
Human complement (HC) is toxic to mouse neurons and oligodendrocytes in monocultures. IncuCyte live imaging of neurons, mature oligodendrocyte (OL), and oligodendrocyte progenitors (OPC) monocultures treated with HC (2% in neurons and 5% in OLs and OPCs) for 4 h. DRAQ7 was added in the culture medium to label dead cells. **a**, **c**, and **e** Phase contrast images show DRAQ7 staining (*red*) in the absence (CTRL) and presence (HC) of human complement. **b**, **d**, and **f** The percentage of DRAQ7+ neurons (**b**), OLs (**d**), and OPCs (**f**) increased rapidly following exposure to HC. **g** and **h** Astrocytes (AST) exposed to 5% HC for 4 h showed no evidence of cytotoxicity as measured and quantified by IncuCyte. CTRL: PBS. Scale bars 100 μm

To investigate the effect of HC on murine CNS cells, we assessed the sensitivity of mouse neurons, oligodendrocytes (OLs), oligodendrocyte progenitor cells (OPCs), and astrocytes to human serum in monocultures. Monocultures were exposed to either PBS as a control or 2–5% (vol/vol) HC. Cell death was measured by the addition of DRAQ7 to the media to label dead/dying cells, followed by IncuCyte live cell imaging. After 4 h of incubation with 2% HC, 90.0 ± 1.9% of the mouse neuronal population was DRAQ7+ (Fig. 1a–b). OLs and OPCs were also sensitive to HC exposure with 70.8 ± 2.8% and 42.2 ± 5.6% cell death, respectively, after 4 h incubation (Fig. 1c–f and Additional file 2: Movie 1 and Additional file 3: Movie 2). In contrast, HC did not induce astrocyte cell death as determined by LDH release assay (Fig. 1g) or IncuCyte live cell imaging (data not shown). The degree of cell death in these murine CNS monocultures in the presence of HC is summarized in Table 1.

**Table 1:** Summary of cell death in monoculture, neuroglial mixed culture and brain slices

Cell death		Monoculture^a^	Mixed culture^b^	Brain slices^c^
Mouse AST	HC	No death	No death	No death
	#53+HC	33.9 ± 1.5%	32.0 ± 1.7%	Damage from 8 h
Mouse N	HC	93.4 ± 4.0%	31.4 ± 4.0%	17.4 ± 0.7%
	#53+HC	97.7 ± 2.1%	72.3 ± 11.1%	52.3 ± 2.7%
Mouse OL	HC	70.8 ± 2.8%	56.0 ± 1.4%	26.4 ± 5.7%
	#53+HC	61.3 ± 7.8%	73.2 ± 5.4%	43.7 ± 3.9%
Mouse OPC	HC	42.2 ± 5.6%	No death	No death
	#53+HC	36.9 ± 5.6%	No death	46.9 ± 16.9%

Abbreviations: *AST* astrocyte, *N* neuron, *OL* mature oligodendrocyte, *OPC* oligodendrocyte precursor cell, *HC* human complement, *#53+HC* rAb #53 plus human complement.

^a^Percentage of cell death at 4 h

^b^Percentage death at 4 h except neuron (N) death with #53+HC which was maximal at 1.5 h

^c^Cell death in brain slices at 48 h



**Movie 1** Pure OL with HC.

**Movie 2** Pure OPC with HC.


### Reduced sensitivity of mouse neurons and OLs to HC in neuroglial mixed cultures

In the CNS, the interaction of neurons and glia may alter their responses to environmental stressors. We investigated how mouse neurons and glial cells responded to HC in mixed cell cultures prepared from PLP-eGFP [20] mouse pups in which both OPCs and differentiated OLs were labeled with eGFP. Cell type specific marker staining showed that the neuroglial mixed cultures consisted of 70% astrocytes (GFAP and AQP4), 10–15% oligodendroglial cells (Olig1), and 10–15% neurons (βIII-Tubulin). Most oligodendroglial cells were OPCs, but occasionally maturing OLs (O4+) were noted (Additional file 1: Figure S1).

**Fig. 2:**
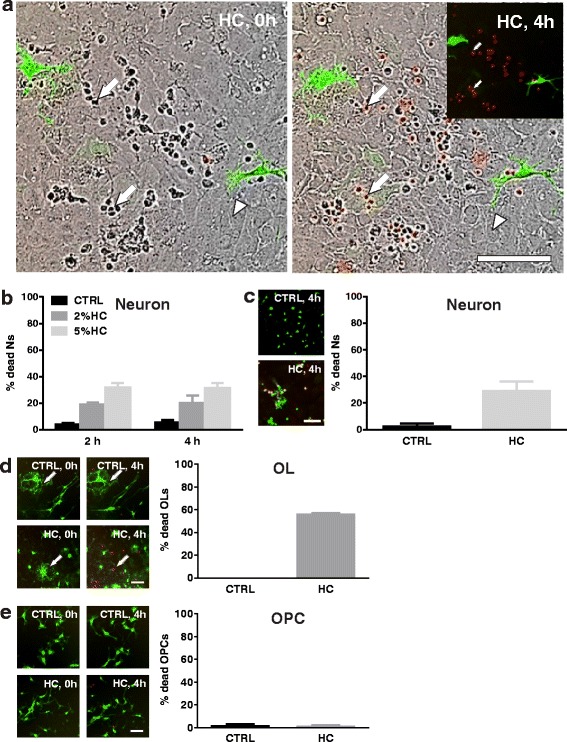
Mixed cultures of neurons, oligodendrocytes, and astrocytes demonstrate reduced complement cytotoxicity. Neuroglial mixed cultures prepared from PLP-eGFP mouse pups were treated with 5% HC for 4 h in the presence of DRAQ7 and imaged using IncuCyte. **a** Phase contrast images of IncuCyte cultures treated with human complement (HC) at 0 and 4 h. *Insert*: Fluorescence image showing oligodendrocytes (PLP-eGFP; *green*) and dead cells (DRAQ7; *red*). *Arrows*: neurons. *Arrow heads*: astrocytes. **b** Quantitation of the percentage of DRAQ7+ neurons at 2 and 4 h in the presence of 2% or 5% HC. **c** Fluorescence image and quantitation of the percentage of PI+ dead neurons following exposure to 5% HC for 4 h. *Left*: fluorescence image of mixed cultures exposed to CTRL or HC and stained with PI (*red*) followed by NeuN (*green*) at 4 h. Note the dead neurons (PI+/NeuN+) in the HC-treated cultures. **d**, **e** IncuCyte fluorescence images (*left*) and quantitation of (**d**) differentiated oligodendrocyte (OL, *arrows*) and (**e**) oligodendrocyte progenitor (OPC) death in mixed cultures. OPCs and differentiated OLs were defined by morphology. More than 150 differentiated oligodendrocytes (OL) were counted for each experiment. CTRL: PBS. Scale bars 100 μm



Cell death in mixed cultures was monitored by IncuCyte live imaging. In the mixed cultures, HC was not toxic to astrocytes (Fig. 2a, arrow heads). Neurons remained sensitive to HC in the mixed cultures (Fig. 2a, arrows); however, the magnitude of loss was significantly reduced. The addition of 5% HC caused 31.4 ± 4.0% neuronal death in the mixed culture after 4 h incubation (Fig. 2b and Additional file 4: Movie 3); whereas, HC resulted in greater than 90% neuronal death in the monoculture (Fig. 1b). Neuronal death was confirmed by co-labeling with the dead cell dye propidium iodide (PI) and neuronal marker NeuN (Fig. 2c).

**Movie 3** Co-culture with HC.


In the neuroglial mixed cultures, the majority of eGFP+ oligodendrocyte lineage cells were bipolar and tripolar progenitors, while a minority of the cells displayed a highly complex network of processes consistent with differentiated OLs [26]. OLs died rapidly in the presence of HC alone with 56.0 ± 1.4% of OLs lost by 4 h (Fig. 2d). OL cell death was reduced, however, in mixed cultures when compared to monocultures (70.8 ± 2.8%, Fig. 1e). In contrast to their behavior in monoculture (Fig. 1d–e), OPCs were not sensitive to HC in the neuroglial mixed cultures, even after an extended incubation time of 3 days (Fig. 2e and Additional file 4: Movie 3). As observed in the monoculture, astrocytes remained insensitive to HC in the neuroglial mixed culture (Fig. 2a and Additional file 4: Movie 3). The percent of CNS cell death in mixed cultures is summarized in Table 1.

### Targeted astrocyte damage increases HC-mediated neuron and OL death in mixed cultures

**Fig. 3:**
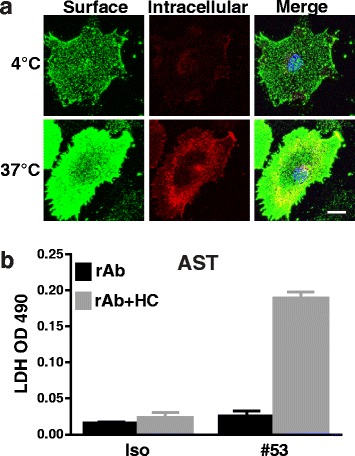
AQP4-specific NMO rAb #53 causes rapid damage to mouse astrocytes in the presence of HC. **a** Differential immunofluorescent labeling of rAb #53 on cell surface and intracellular compartments in live astrocyte cultures at 4 °C or 37 °C. **b** LDH activity was measured in the clarified cell-free culture supernatant recovered from the astrocyte monoculture treated with rAbs #53 with or without 5% HC for 4 h at 37 °C. Iso: negative isotope control rAb. Scale bars 20 μm

We hypothesized that astrocytes in mixed cultures provided protection against HC to adjacent oligodendrocytes and neurons. To test this, we used a recombinant monoclonal antibody specific for AQP4 (rAb #53) [12] to target astrocytes via complement-mediated lysis (CDC) [13]. Live staining of rAb #53 of astrocytes at 4 or 37 °C displayed a robust membrane staining and dim cytoplasmic staining, indicating only a small portion of AQP4 being internalized from the cell surface (Fig. 3a). In combination with HC, rAb #53 induced significant astrocyte death, resulting in a marked increase of LDH in the culture medium (Fig. 3b).

**Fig. 4:**
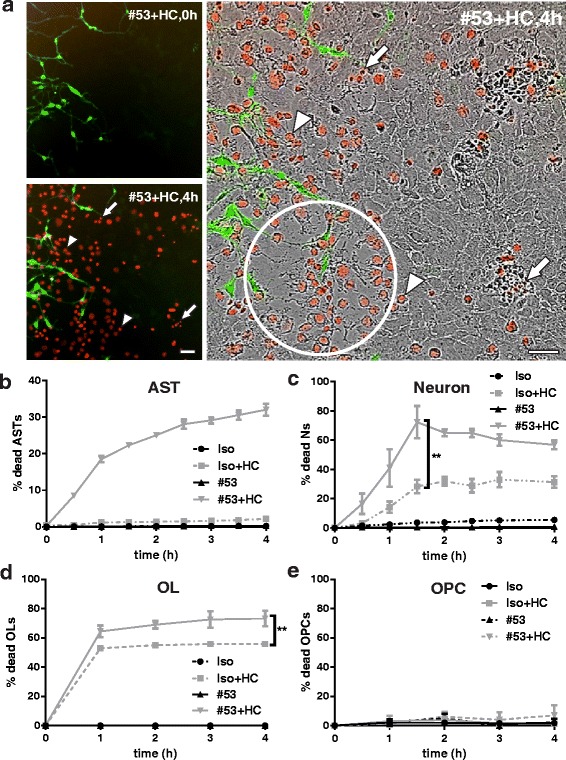
Kinetics of neuron, OL, and OPC loss in the presence of NMO rAb #53-mediated astrocyte injury. **a** Fluorescence (left) and phase contrast IncuCyte (right) images of neuroglial mixed culture treated with rAb #53 plus 5% HC in the presence of DRAQ7 (PLP-eGFP oligodendrocytes [green] and DRAQ7 dead cells [red]). *Arrows*: neurons. *Arrow heads*: astrocytes. *Circled area*: focal region of astrocyte depletion. **b**, **c**, **d**, and **e** Quantitation of the percentage of DRAQ7+ AST (**b**), neuron (**c**), OL (**d**), and OPC (**e**) in the mixed cultures. Cells were identified as described in the Methods. Statistical analyses were performed by multiple unpaired Student’s *t* test. **p < 0.01, *n* > 3. Scale bars 50 μm



We monitored the kinetics of neuron, oligodendrocyte, and astrocyte death in the mixed cultures following targeted rAb #53-mediated astrocyte damage with IncuCyte live cell imaging. No cell death was noted with rAb #53 or isotype control (Iso) without the presence of HC. Treatment with rAb #53+ 5% HC induced rapid cell death, notably among astrocytes (Fig. 4a–b). Dead neurons were identified as small round DRAQ7+ particles with high fluorescence intensity (Fig. 4a, arrows), whereas dead astrocytes were distinguished as larger but less intense DRAQ7+ cells (Fig. 4a, arrow head). Dead astrocytes floated into the culture supernatant, resulting in areas of astrocyte depletion (Fig. 4a, circled area). After the peak of astrocyte death (4 h), viable astrocytes repopulated these areas (Additional file 5: Movie 4). Neuronal death increased in the presence of rAb #53-mediated astrocyte destruction, with approximately 70% of neurons dying within 2 h. While some neuronal death may result from their sensitivity to HC alone, only 30% neuronal death was observed with Iso+HC (Fig. 4c) or HC alone (Fig. 2b–c). Astrocyte loss induced by rAb #53 plus HC also increased the death of OLs (Fig. 4d). In contrast, rAb #53-mediated astrocyte destruction caused no loss of OPCs (Fig. 4e). The percent cell death is summarized in Table 1.

**Movie 4** Co-culture with NMO53+HC.


The sensitivity of neurons, OL, and astrocytes to HC was not dependent on the source of human sera. Both pooled and single donor samples of HC from different vendors were tested for cytotoxic activity on mixed neuroglial cultures and produced identical cytotoxic effects (Additional file 1: Figure S2 and Table S1). Consistent with previous reports [18, 19], we did not detect any cytotoxic effects of mouse serum on neurons or OLs in mixed culture in the presence or absence of AQP4-specific rAb #53 (Additional file 1: Figure S2 and Table S1).

### Sensitivity to HC is further modified and attenuated in mouse organotypic cerebellar slices

**Fig. 5:**
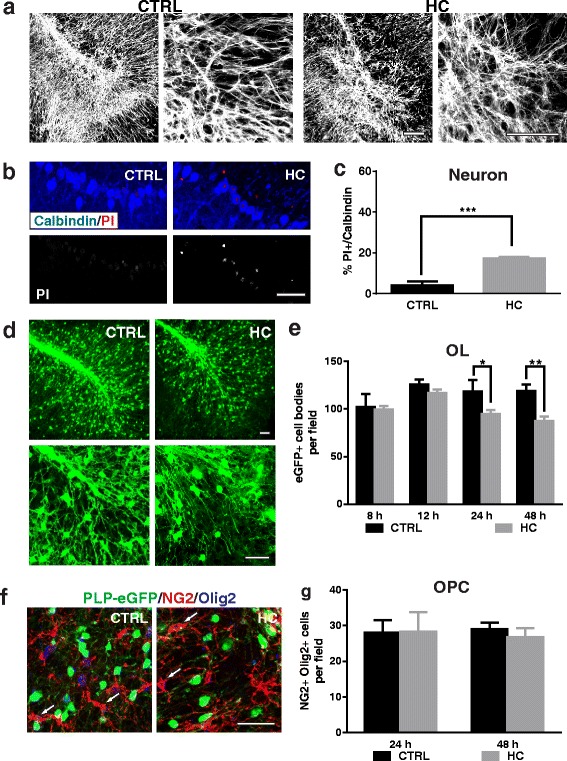
HC causes mild neuronal and mature oligodendroglial cell loss in mouse organotypic cerebellar slice culture. Immunostaining and quantitation of neuron and glia cell death in the slices treated with medium only (CTRL) or 10% HC for 48 h. **a** 25× (*left panels*) and 63× (*right panels*) objective images of slices stained with AST marker GFAP. **b**, **c** Calbindin-positive Purkinje neurons show increased PI staining in the presence of HC. **d**, **e** PLP-eGFP-positive OLs are mildly reduced in number following HC treatment for 48 h. 25× (*upper*) and 63× (*lower*) objective images of PLP-eGFP in slices treated for 48 h (**d**). eGFP+ cell bodies were quantified at 8, 12, 24, and 48 h in multiple 20× fields (**e**). **f**, **g** Slices were treated for 48 h and co-stained with Olig2 and NG2. *Arrows* mark Olig2+NG2+ OPCs. OPCs were counted at 24 or 48 h following HC administration. Statistical analyses were performed by multiple unpaired Student’s *t* test. *p < 0.05, **p < 0.01, *n* ≥ 3. Scale bars 50 μm

In order to determine the sensitivity of CNS cells to HC in a complex tissue network, cell death was examined in mouse organotypic cerebellar slices. Slices treated with HC alone had no effect on astrocyte morphology and tissue structure (Fig. 5a). A low level of neuronal death was detected at 48 h following HC treatment: 17.4 ± 0.7% of calbindin-positive neurons were PI-positive (Fig. 5b–c). Neuronal death, however, was not limited to Purkinje cells, as coincident NeuN and PI staining showed a general loss of neurons throughout the slice, including the granule cell layer (Additional file 1: Figure S3).

In cerebellar slice cultures, all PLP-eGFP positive cells differentiated into mature oligodendrocytes and co-localized with CC1 (data not shown), a mature oligodendrocyte marker [27]; OPCs no longer expressed eGFP [20]. HC caused a significant loss of PLP-eGFP+ cell bodies from 24 h treatment. At 48 h, the number of eGFP+ oligodendrocyte cell bodies was reduced by 25% with some PLP-eGFP+ OLs displaying disorganized processes (Fig. 5d–e). OPCs were visualized with NG2 and Olig2 co-staining and did not express PLP-eGFP (Fig. 5f, arrows). No loss of OPCs was observed in cerebellar slices treated with HC alone for 48 h (Fig. 5f–g).

**Fig. 6:**
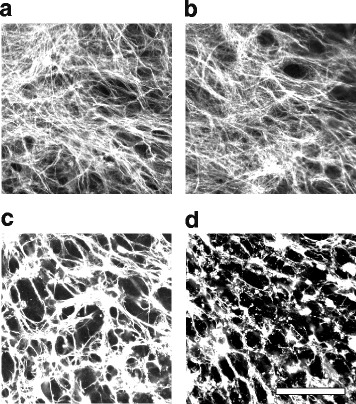
NMO rAb #53 and HC cause rapid astrocyte network destruction in mouse brain slices. Images of slices treated with rAbs #53 and isotope control rAb with or without 10% HC for 8 or 48 h and stained with GFAP. **a** rAb #53 alone, 48 h. **b** rAb Iso with HC, 48 h. **c** rAb #53 with HC 8 h. **d** rAb #53 with HC, 48 h. Iso: negative isotype control rAb. Scale bar 50 μm

**Fig. 7:**
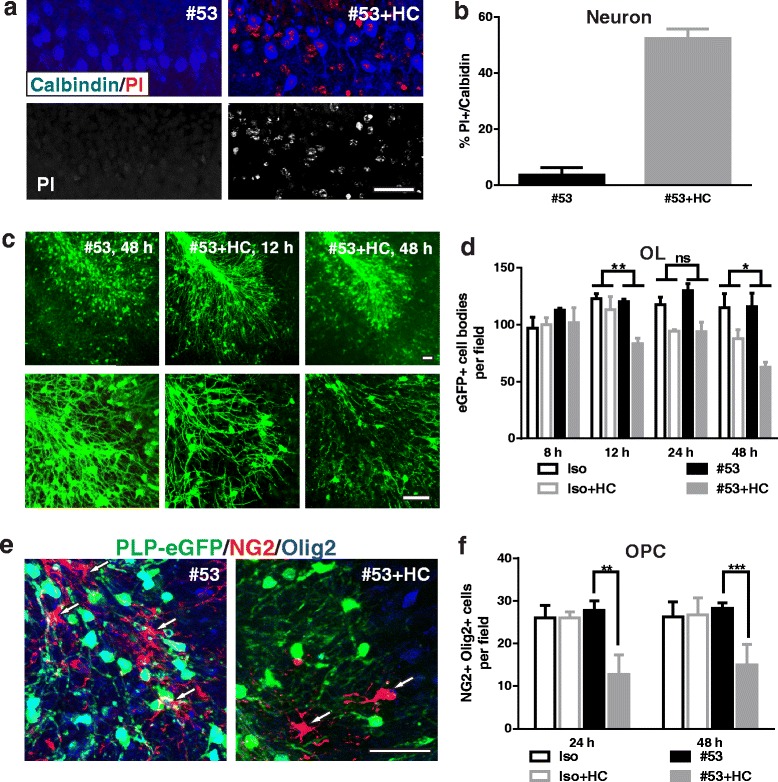
Increased neuron and OL death following astrocyte damage in brain slices. Immunostaining and quantification of neuron and oligodendrocyte cell death in the slices treated with rAb NMO#53 or isotype control rAb with or without 10% HC for 48 h. PI-stained cells double stained with Purkinje neuron marker Calbindin (**a**). Quantification of PI-labeled cells co-localized with Calbindin, as a percent of the total Calbindin+ cells (**b**). 25× (*upper panel*) and 63× (*lower panel*) objective images of PLP-eGFP in slices (**c**). Quantification of eGFP+ OLs in slices at 8, 12, 24, and 48 h after treatment (**d**). Cerebellar slices treated for 24 h with rAb #53 and HC were co-stained with Olig2 and NG2 to identify OPCs (**e**). *Arrows* mark OPCs. **f** OPCs were counted in slices treated for 24 and 48 h. Iso: negative isotope control rAb. Statistical analyses were performed by multiple unpaired Student’s *t* test for single comparison (**f**) or by two-way ANOVA for grouped comparisons (**d**). *p < 0.05, **p < 0.01, ***p < 0.001, ns not significant, *n* ≥ 3. Scale bars 50 μm

To further investigate the role of astrocyte injury on cell-specific survival within organotypic cultures, we treated cerebellar slices with NMO rAb #53+HC. In rAb #53+HC-treated slices, astrocytes became swollen, and the network of astrocytic processes was disrupted within 8 h. At 48 h, massive destruction of the astrocyte network was apparent (Fig. 6c–d). In contrast, no significant morphological changes in astrocytes were detected using an isotype control rAb+HC or rAb #53 alone (Fig. 6a–b). At 48 h, neuronal death in the slices treated with rAb #53+HC increased about threefold compared to HC alone: 52.3 ± 2.7% calbindin-positive neurons were PI-positive following rAb #53+HC (Fig. 7a–b), as compared with 17.4 ± 0.7% with HC alone (Fig. 5b–c). With increasing astrocyte destruction, the loss of OLs rapidly escalated: approximately 30% of OLs were lost at 12 h, whereas, at 48 h, about 50% of OLs were lost (Fig. 7c–d). The loss of OLs at 48 h was increased approximately 1.6-fold compared to HC alone (Fig. 5d–e). Surprisingly, approximately 50% of NG2+ Olig2+ OPCs were lost in slices by 24 h after treatment with rAb #53+HC (Fig. 7e–f, arrows), despite their lack of sensitivity to HC in neuroglial mixed cultures following astrocyte loss (Fig. 4e and Additional file 5: Movie 4) and their resistance to HC alone in organotypic cultures. The results are summarized in Table 1.

### Sensitivity of murine cells to HC is due to cross-reactive IgGs and requires complement activation

**Fig. 8:**
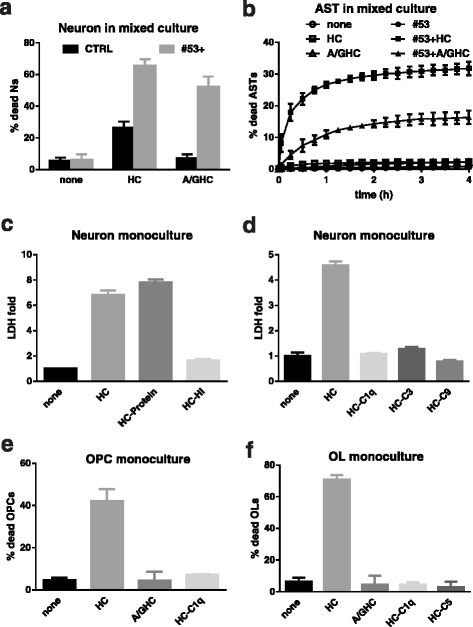
Anti-tissue antibodies and complement proteins in the human serum were required in the cytotoxicity to neurons, OPCs and OLs. **a** IgG-depleted human serum by protein A/G agarose (A/GHC) were used in mixed culture and assayed for neuronal death by IncuCyte. Unlike HC, A/GHC did not cause significant neuronal death. **b** With addition of NMO rAb #53, A/GHC caused astrocyte death at a reduced level compared with #53+HC. **c**, **d** Serum protein or heat-inactivated human serum (**c**) or human serum with depletion of complement components were used in neuron monoculture cytotoxicity assay (**d**). **﻿﻿﻿e**, **f** Human se﻿rum (HC), or serum depleted of IgG by A/G agarose (A/GHC) or complement ﻿C1q (HC-C1q), were used in OPC (**e**) or OL (**f**) monoculture cytotoxicity assays. ﻿﻿﻿LDH release was measured in the clarified cell-free supernatant collected from cultures treated as indicated for 4 h. HC-protein: protein fractions of human serum retained from 3KD centrifugal filters, HC-HI: heat-inactivated human serum, HC-C1q: C1q-depleted human serum, HC-C3: C3-depleted human serum, and HC-C9: C9-depleted human serum

Because of the intrinsically weak activity of mouse complement [18] and the presence of complement inhibitor(s) in the mouse serum [19], complement sourced from human serum is required to trigger complement activation in NMO models. Our results demonstrate that murine neurons and oligodendroglia are sensitive to HC in monocultures and mixed cell cultures. Human neurons, however, did not demonstrate a similar sensitivity to HC (Additional file 1: Figure S4). Similarly, rat OPCs and OLs showed no sensitivity to rat serum (Additional file 1: Figure S4). These data suggest that human serum may contain cross-reactive antibodies that are mediating murine neuron and oligodendrocyte cell death. Using heat inactivation, which eliminated complement protein activity but maintained human antibody function, we observed binding of HC-derived human IgG to murine neurons, OLs, OPCs, and astrocytes (Additional file 1: Figure S5). Furthermore, IgG-depleted HC was not toxic to mouse neurons, OPCs or OLs in the monocultures or in mixed cell culture, and retained its ability to induce CDC on cultured astrocytes in the presence of rAb #53 (Fig. 8a–b and e–f). To determine if the complement pathway is required for HC-mediated cell death of murine cells, we heat inactivated or depleted HC of complement components (C1q, C3, C5, or C9) and applied it to murine monocultures. Inactivation of the complement pathway prevented HC-mediated cell death of murine cells (Fig. 8c–f). Taken together, these results suggest cross-reactive antibodies in human sera bind to the surface of mouse cells and may activate the complement cascade.

**Fig. 9:**
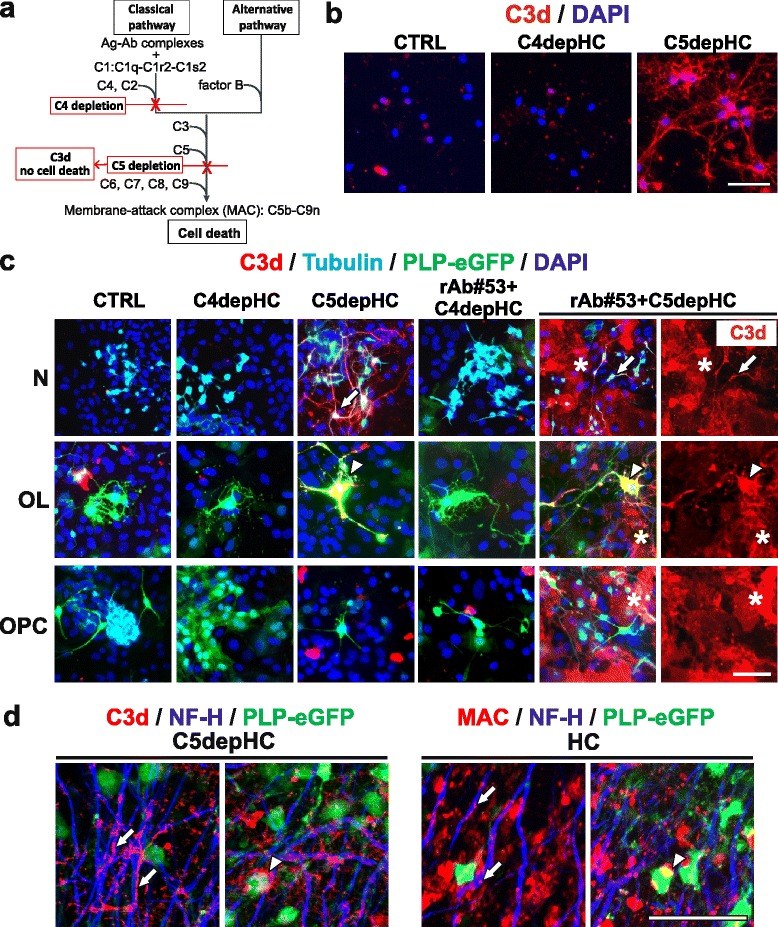
HC toxicity to mouse CNS cells requires activation of the classical complement cascade. **a** Simplified diagram of the complement cascade depicting both the classical and alternative pathways. *Red lines* indicate the location of blocks in the complement pathway resulting from either C4 or C5 depletion. **b** C3d staining in pure neuronal monocultures. Cultures were treated with medium only (CTRL), 5% C4-depeleted, or C5-depleted human complement (C4depHC or C5depHC) for 30 min followed by live staining with anti-C3d antibody and DAPI. **c** C3d staining in neuroglial mixed cultures. Mixed cultures prepared from PLP-eGFP mouse pups were incubated with medium only (CTRL), 5% C4depHC, or 5% C5depHC in the presence/absence of NMO rAb #53 for 30 min followed by live staining of C3d. Cells were then fixed and stained with neuronal marker β-Tubulin and DAPI. Note: In mixed cultures treated with C5depHC alone, C3d was present on the surface of neurons (N, *arrows*) and differentiated OLs (OL, *arrow heads*), which were sensitive to HC. In cell cultures treated with NMO rAb #53 plus C5depHC, C3d was detected on the surface of neurons (*arrows*), OLs (*arrow heads*), and surrounding astrocytes (*stars*). No C3d staining was observed on OPCs in mixed cultures. **d** C3d and MAC staining in slice culture. PLP-eGFP slices were treated with 10% C5depHC or human complement (HC) for 48 h and stained with C3d and MAC, respectively. Neurofilament was visualized with NF-H staining. Deposits of C3d or MAC were detected along the NF-H+ processes (*arrows*) and surrounding the oligodendrocyte cell bodies (*arrow heads*). Scale bars 50 μm

Complete activation of the complement cascade leads to the formation of the terminal membrane attack complex (MAC) and cell lysis. To determine which cells are targeted for MAC formation, we used selective depletion of proteins along the classical and common pathways to prevent cell death and localize the deposition of activated complement components. Using C5-depleted HC, we arrested complement activation following C3 proteolytic cleavage resulting in C3d deposition on complement-targeted cells (Fig. 9a). In neuronal monocultures treated with C5-depleted HC, C3d was detected on the cell surface. When neuronal monocultures were treated with C4-depleted human complement (Fig. 9b), there was no C3d deposition, indicating that activation of the classical pathway was essential for neuronal injury with HC alone. In neuroglial mixed cultures treated with C5-depleted HC, C3d was detected on the surface of neurons and OLs (arrows and arrow heads, respectively), but not OPCs or surrounding astrocytes (Fig. 9c). Since HC-derived IgG bound to murine neurons, OLs, OPCs, and astrocytes (Additional file 1: Figure S5), the resistance of OPCs and astrocytes to CDC in mixed culture was either due to the local expression of complement inhibitors or the inability of surface-bound human IgGs to reach a sufficient threshold to activate the complement cascade. When mixed cultures were treated with NMO rAb #53 and C5-depleted HC, C3d deposits were found on the surface of neurons (arrows), OLs (arrow heads), and surrounding astrocytes (stars), but not on OPCs (Fig. 9c). In organotypic cerebellar slices treated with C5-depleted or complete HC, deposits of C3d and MAC were detected along fibers containing neuro-filaments (Fig. 9d, arrows) and surrounding oligodendrocyte cell bodies (Fig. 9d, arrowheads). No detectable C3d or MAC deposits were noted in slices exposed to medium alone (data not shown). These results demonstrate that complement in human serum is activated by human anti-murine cross-reactive antibodies in primary cell monocultures, mixed cultures, and cerebellar slice cultures.

## Discussion

These studies demonstrated that activated human complement HC is injurious to mouse neurons and OLs in a variety of culture conditions. We found that complement activation is necessary for cell injury in primary cell and slice cultures and occurs through the binding and activation of the classical pathway by anti-murine immunoglobulin in human sera. The susceptibility of neurons and glia to HC in the culture system lessens as the CNS cells coalesce into more complex networks. It may be that the different culture conditions (i.e., mono- or mixed cell cultures or tissues) alter the expression profiles of individual cell types resulting in loss of antigen expression, masking of target epitopes, and/or acquisition of complement inhibition. Each of these mechanisms may play a role in cell injury in models of NMO lesions and need to be examined relative to pathology in human tissue.

In primary cell monocultures, the addition of human serum alone is sufficient to cause rapid and substantial oligodendrocyte and neuronal cell death. In the neuroglial mixed cultures, the effects of activated complement are likely reduced through protective oligodendroglial and neuronal interactions with astrocytes. As a consequence, astrocyte injury and loss driven by AQP4-specific rAb and complement-mediated cytotoxicity rapidly increase the level of neuronal and oligodendroglial damage. Interestingly, OPCs in the neuroglial cultures are resistant to complement attack following astrocyte damage. In cerebellar tissue slices, cell-cell interactions are more complex, and complement-mediated cell death of neurons and OLs are further reduced. While astrocyte destruction similarly increases oligodendroglial and neuronal cell death, the susceptibility of these populations to complement cytotoxicity independent of astrocyte loss is much less than that observed in cell cultures. OPCs are the notable exception, becoming sensitized in the presence of astrocyte damage in organotypic slice culture (Table 1). Our results suggest that the increasing complexity of the neuroglia environment, particularly in slice cultures, protects neurons and oligodendrocytes from complement-induced cytotoxicity, and astrocytes play a central role in modulating complement injury. In the CNS of NMO patients, additional factors other than CDC, such as excitotoxicity, inflammatory cytokines, antibody-dependent cell-mediated cytotoxicity, anaphylaxotoxins, and disrupted astrocyte physiology may play critical roles in lesion formation (reviewed in [28]). Whether exposure of tissue epitopes allows CDC to contribute to oligodendrocyte and neuronal injury in human NMO lesions remains to be determined. Conversely, the use of complement from multiple species will be necessary to validate the relevance of oligodendroglial and neuronal damage observed across in vitro, ex vivo, and in vivo NMO models.

Astrocytes are a major producer of complement in the healthy and diseased CNS [29]. While astrocytes are not sensitive to HC alone, astrocyte destruction occurs readily through the production of terminal complement complexes (MAC complex) with AQP4-specific antibodies. Indeed, the current and previous studies demonstrate that inactivation of the classical or alternative pathway affects AQP4-IgG mediated astrocyte cytotoxicity [16, 30]. Antibodies against factor B [31], a key regulator of alternative pathway, or HC depleted of factor B, decrease astrocyte damage [16].

Neurons in monoculture were very sensitive to activated complement, in agreement with the previous observations that neurons, unlike most peripheral cell types, express complement receptors and regulators, but do not express the most known complement inhibitors [32, 33]. Neuronal damage in our in vitro and ex vivo models occurred as the direct result of activated complement. This may, in part, explain why persistent neurologic disability is strongly linked with acute relapses, as activated complement is prominent feature of acute NMO lesions [1, 34, 35].

Consistent with previous reports [36], our results demonstrated that oligodendrocytes were vulnerable to activated complement. Both in cell culture and brain slices, OLs (O4+) were susceptible to the addition of HC independent of the presence of astrocytes. Oligodendrocytes, unlike astrocytes, lack the complement regulatory protein CD59, which inhibits the final formation of membrane attack complex [37–39]. Complement resistance in rat oligodendrocytes can be restored by the incorporation of purified rat CD59 into the cell membrane. Furthermore, neutralization of rat CD59 on complement-resistant astrocytes rendered them susceptible to CDC [37]. When studied in mixed cultures or slice cultures with astrocytes and neurons, OL susceptibility to complement was partially decreased (Table 1). One explanation is that the presence of astrocytes or neurons may reduce the amount of the activated complement available for OL binding, mask OL membrane epitopes, or alter OL membrane epitope expression. Alternatively, astrocytes or neurons may secrete, exchange, or modulate complement regulatory protein expression on the oligodendrocyte surface. It has been reported that in a mixed culture system of rat oligodendrocytes and dorsal root ganglion neurons, OLs are remarkably less sensitive to complement. Incubation of OLs with neuron-conditioned medium afforded a similar protection against complement-mediated lysis [40].

Our results demonstrate that OPCs remain resistant to activated complement independent of the astrocyte damage in mixed cultures. Unexpectedly, in organotypic cerebellar slice cultures, astrocyte destruction enhanced OPC loss. Although the neuroglia mixed cultures are prepared from the cortex and the organotypic slices from cerebellum, the differential sensitivity of OPCs to astrocyte damage does not appear to result from distinctions between cortical and cerebellar glia. In cell cultures prepared from cerebellum, there are no significant differences in astrocyte death or OPC survival in the presence of NMO rAb #53 and HC (unpublished data). Thus, it appears that astrocyte damage in the complex neuroglia network of cerebellar slices sensitizes OPCs to injury. In addition, activated microglia, inflammatory mediators, and excitotoxic molecules may combine to produce a toxic milieu for OPCs in brain slices. Alternatively, the state of differentiation of OPCs in that environment may result in novel susceptibility to neuroinflammatory injury.

Following astrocyte damage, loss of oligodendrocytes, both mature cells and progenitors, is observed earlier than neuronal death in cerebellar slices. This is consistent with reports that the loss of OLs and OPCs are early pathologic features after astrocyte depletion in human and experimental NMO lesions [41]. Vulnerability of oligodendrocytes is found in many neurological conditions. In patients with relapsing-remitting multiple sclerosis (RRMS), oligodendrocytes in affected tissue show apoptotic features and myelin sheaths stain with activated complement [42]. In a rat model of hypoxia-ischemic injury and in a mouse model of amyotrophic lateral sclerosis (ALS), extensive degeneration of differentiated oligodendrocytes (O4+) is found before the loss of OPCs [43, 44]. Although new oligodendrocytes are formed from NG2+ OPCs in the spinal cord of ALS mice, they fail to mature [44]. These observations in combination with our results suggest a selective vulnerability of mature oligodendrocytes to multiple forms of injury. With extreme conditions, such as massive astrocyte destruction in NMO, OPCs may also be affected.

Taken together, our findings demonstrate that in mouse monoculture, activated human complement is targeted to neurons, OLs and OPCs through the action of anti-murine immunoglobulins. In mixed cultures, surrounding astrocytes ameliorate CDC; targeted destruction of astrocytes with AQP4-specific recombinant monoclonal antibodies restores neuronal and oligodendroglia susceptibility to HC. Increasing complexity of neuroglial networks in murine cerebellar slices further reduces the effects of activated human complement alone and alters the secondary effects of astrocyte injury on OPC survival. Experimental models of NMO require complement activation to initiate CNS lesions. Initial models involved administration of AQP4-IgG to rats with pre-existing neuroinflammation produced by experimental autoimmune encephalomyelitis [12, 14, 45]. Although some pathology resulted from the influx of myelin-reactive T cells, lesions demonstrated hallmark features of NMO pathology including perivascular astrocyte destruction and complement deposition. Subsequent passive-transfer models of NMO in mice required intracranial injection of NMO-IgG and human complement to recapitulate key pathological findings in human lesions [30, 46, 47]. The use of human complement was required due to the presence of inhibitors in mouse complement preparations [18, 19]. The current studies indicate that one should use caution in interpreting the cause of secondary injury to CNS cells following AQP4-IgG mediated CDC when using human serum in murine models.

## Conclusions

Our study demonstrates that mouse neurons, OLs, and OPCs display variable sensitivity to activated HC based on culture conditions and tissue complexity. In cell and slice cultures, the protection of neurons, OLs, and OPCs against HC is eliminated by targeted astrocyte destruction. Because of the targeted activation of HC on mouse CNS cells, caution is needed in interpreting the relationship between damage in experimental mouse models of NMO and human lesions.

## Additional files

Additional file 1: Figure S1. Immunostaining with cell type specific markers to identify different cell types in the neuroglial co-cultures. **Figure S2.** Cytotoxic effects of human and mouse complement on neuroglial mixed cultures. **Figure S3.** Confocal images of PI-stained cells double stained with neuronal marker NeuN in slices treated with 10% HC or rAbs (NMO#53) plus HC for 48 h in slices. **Figure S4.** Human neurons and rat OPCs and OLs were not sensitive to human serum or rat serum. **Figure S5.** Binding of HC-derived human IgG to the surface of murine neurons, OLs, OPCs and astrocytes. **Table S1.** Cytotoxic effects of multiple sources of human (HC) and mouse complement (MC) in neuroglial mixed cultures. (PDF 648 kb)

## Abbreviations

AQP4: Aquaporin-4
AQP4-IgG: Aquaporin-4 autoantibody
AST: Astrocyte
BSA: Bovine serum albumin
C4-C5depHC: C4 or C5 depleted human serum
CDC: Complement-dependent cytotoxicity
CNS: Central nervous system
CSF: Cerebrospinal fluid
CTRL: Control
eGFP: Enhanced green fluorescent protein
FBS: Fetal bovine serum
FGF: Fibroblast growth factor
GFAP: Glial fibrillary acidic protein
HC: Human complement
IgG: Immunoglobulins
LDH: Lactate dehydrogenase
MAC: Membrane attack complex
NDS: Normal donkey serum
NF-H: Neurofilament heavy chain
NGS: Normal goat serum
NMO: Neuromyelitis optica
OL: Oligodendrocytes
OPC: Oligodendrocyte progenitors
PBS: Phosphate-buffered saline
PDGF: Platelet-derived growth factor
PI: Propidium iodide
PLP: Proteolipid protein
rAb: Recombinant antibody
