# Supplementary material for: Variable sensitivity to complement-dependent cytotoxicity in murine models of neuromyelitis optica
Source: J Neuroinflammation. 2016 Dec 1;13:301. doi: 10.1186/s12974-016-0767-4 (PMC5134246; doi:10.1186/s12974-016-0767-4)
Supplement: Additional file 1: Figure S1. — Immunostaining with cell type specific markers to identify different cell types in the neuroglial co-cultures. Figure S2. Cytotoxic effects of human and mouse complement on neuroglial mixed cultures. Figure S3. Confocal images of PI-stained cells double stained with neuronal marker NeuN in slices treated with 10% HC or rAbs (NMO#53) plus HC for 48 h in slices. Figure S4. Human neurons and rat OPCs and OLs were not sensitive to human serum or rat serum. Figure S5. Binding of HC-derived human IgG to the surface of murine neurons, OLs, OPCs and astrocytes. Table S1. Cytotoxic effects of multiple sources of human (HC) and mouse complement (MC) in neuroglial mixed cultures. (PDF 648 kb) [file 12974_2016_767_MOESM1_ESM.pdf]

Supporting Information

Movies:

Movie 1: Pure OL with HC

Movie 2: Pure OPC with HC

Movie 3: Co-culture with HC

Movie 4: Co-culture with NMO53+HC

Movie 1: Pure OL with HC. IncuCyte live cell imaging in purified differentiated OL mono-culture treated with 5%HC. Cell cultures were scanned for 24hr. Images were captured at 15 min intervals with high definition phase contrast and red fluorescence (to detect the dead cell nuclei dye DRAQ7).

Movie 2: Pure OPC with HC. IncuCyte live cell imaging in purified OPC mono-culture treated with 5%HC. Cell cultures were scanned for 24hr. Images were captured at 15 min intervals with high definition phase contrast and red fluorescence (to detect the dead cell nuclei dye DRAQ7).

Movie 3: Co-culture with HC. IncuCyte live cell imaging in neuro-glial co-cultures prepared form PLP-eGFP mouse pups and treated with 5%HC. Cell cultures were scanned for 66hr. Images were captured at 15 min intervals with high definition phase contrast, green (to detect eGFP+ OPC and differentiated OL) and red fluorescence (to detect the dead cell nuclei dye DRAQ7).

Movie 4: Co-culture with NMO53+HC. IncuCyte live cell imaging in neuro-glial co-cultures prepared form PLP-eGFP mouse pups and treated with NMO rAb#53 plus 5%HC. Cell cultures were scanned for 66hr. Images were captured at 15 min intervals with high definition phase contrast, green (to detect eGFP+ OPC and differentiated OL) and red fluorescence (to detect the dead cell nuclei dye DRAQ7). Around the area marked with “A”: dead astrocytes floated into the culture supernatant, resulting in focal areas of astrocyte-depletion (also shown in Figure 4a, circled area). After the peak of astrocyte death, astrocytes repopulated the depleted areas.

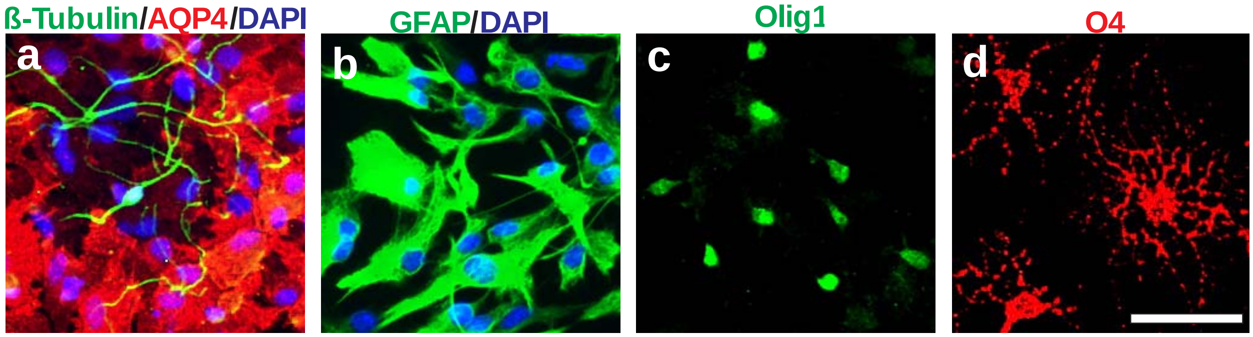

Figure S1: Immunostaining with cell type specific markers to identify different cell types in the neuro-glial co-cultures. Neuro-glial co-cultures were stained with: (**a**) neuronal marker β-Tubulin, astrocyte marker AQP4, and DAPI; (**b**) astrocyte marker GFAP and DAPI; (**c**) oligodendrocyte lineage cell marker Olig1; (**d**) differentiated oligodendrocyte cell marker O4. Scale bar: 50µm.

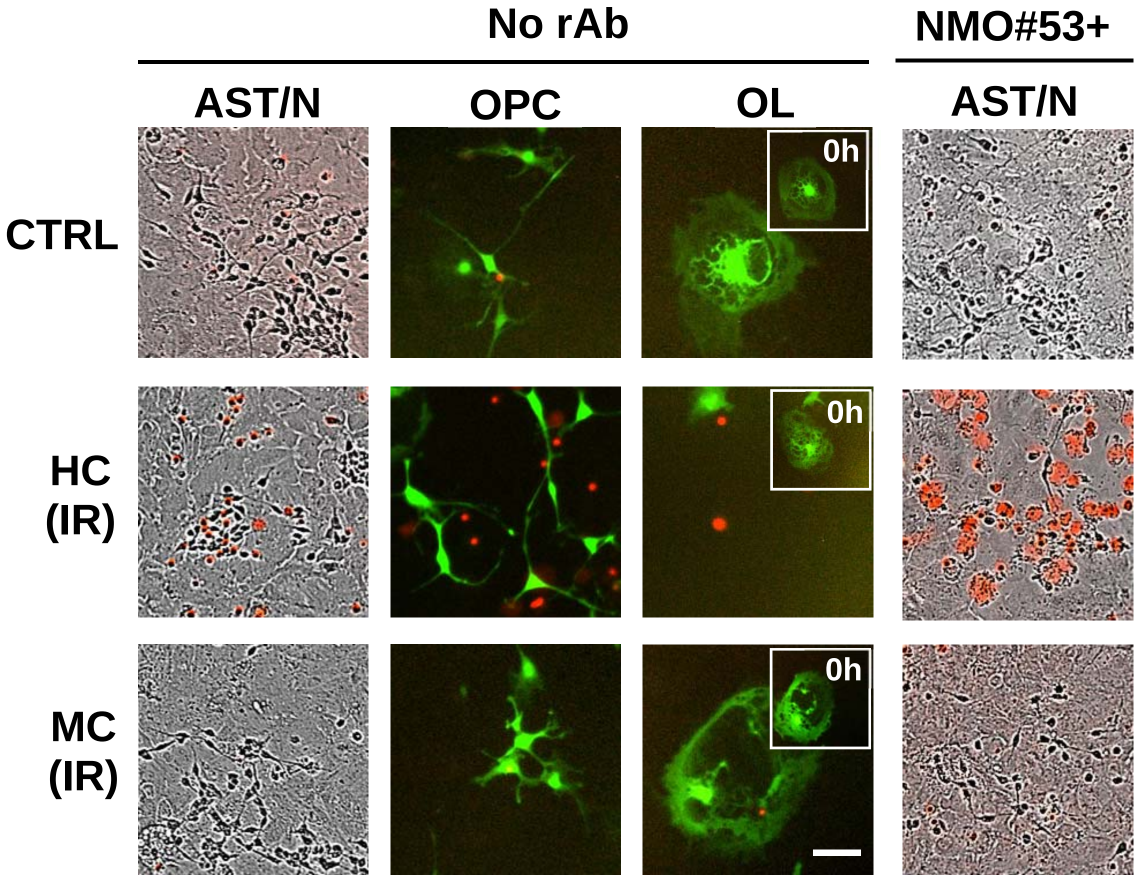

Figure S2: Cytotoxic effects of human and mouse complement on neuro-glial mixed cultures. Representative Incucyte phase contrast (AST/N) and fluorescence (OPC and OL) images of neuro-glial mixed cultures treated with human complement (HC) or mouse complement (MC) with or without NMO rAb#53 for 4 hours. 5 different human sera and 2 different mouse sera were tested. DRAQ7 was added in the culture medium to label dead cells (red) which were classified as neurons (N) or astrocytes (AST) based on signal intensity and morphology. The addition of HC alone caused neuron death (left panel, small red dots). In the presence of NMO rAb#53, HC induced cell death of astrocytes (right panel; low intensity, large red dots) and neurons (right panel, small red dots). Oligodendrocytes (OL) were sensitive to HC, and oligodendrocyte precursors (OPC) were resistant to HC. The inserts depict mature PLP-eGFP+ OLs at experimental onset. MC, with or without the addition of NMO rAb#53, did not result in any cytotoxic effects on neurons, astrocytes, OPCs or OLs. Pooled human and mouse sera were from Innovative Research. Scale bar: 50μm.

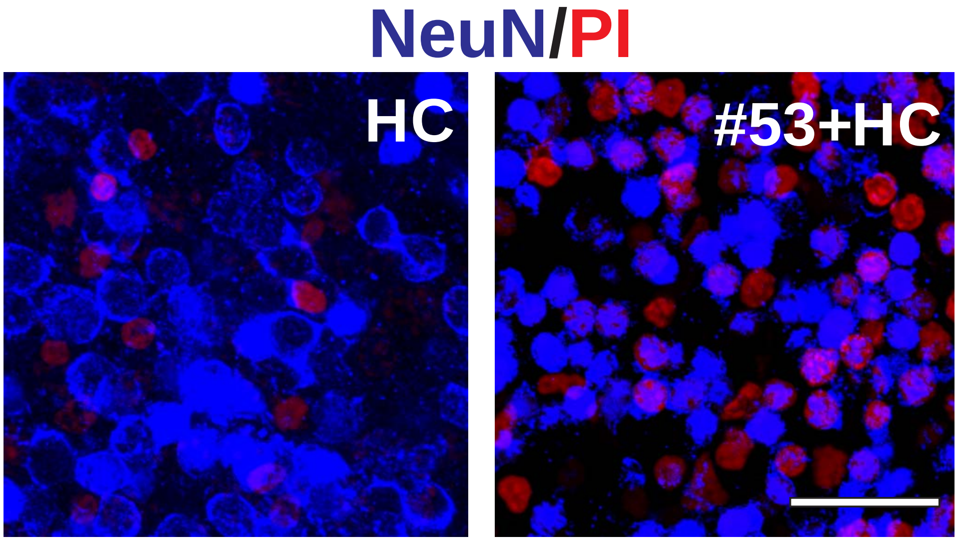

Figure S3: Confocal images of PI-stained cells double stained with neuronal marker NeuN in slices treated with 10% HC or rAbs (NMO#53) plus HC for 48 h in slices. Scale bar: 50µm.

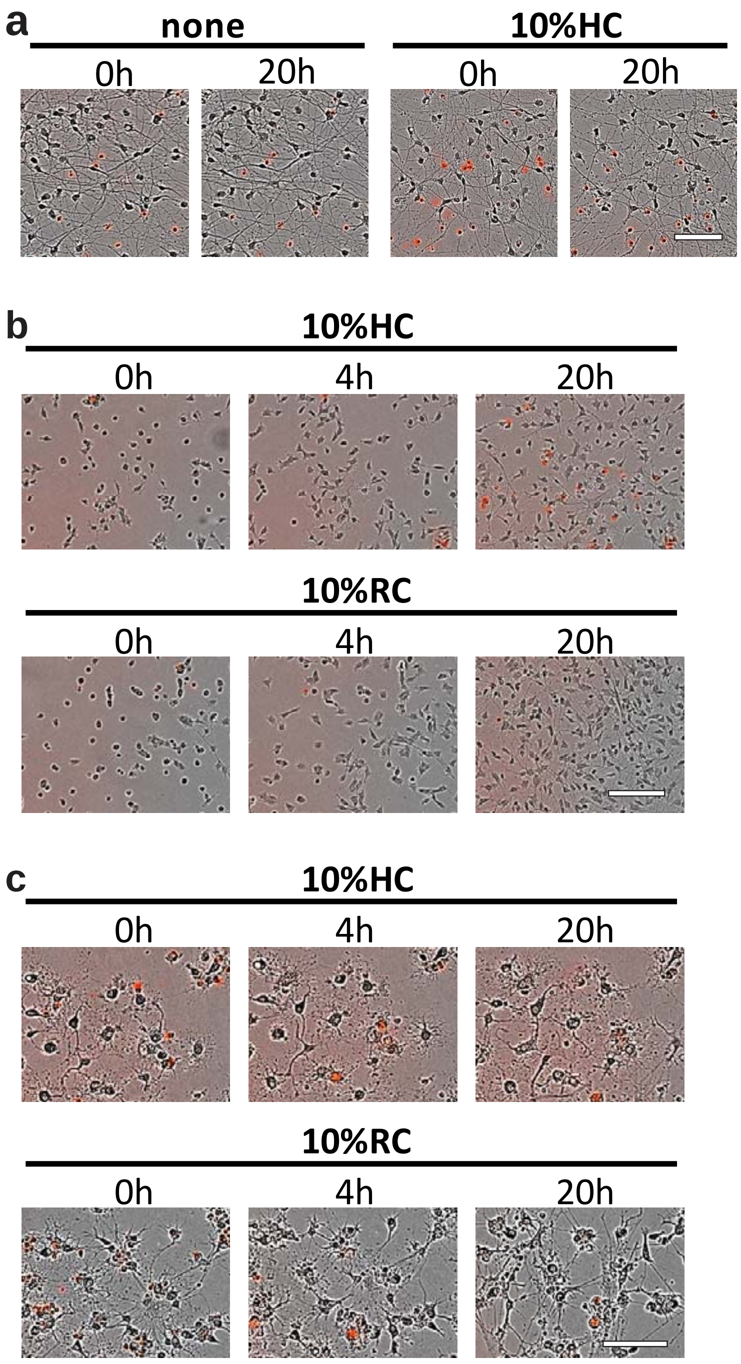

Figure S4: Human neurons and rat OPCs and OLs were not sensitive to human serum or rat serum. IncuCyte live imaging of (a) Human cortical neuron cultures (iCell Neurons), (b) rat OPC, and (c) rat mature OL monocultures treated with10% human serum as the source of human complement (HC) or 10% rat serum as source of rat complement (RC). DRAQ7 was added in the culture medium to label dead cells. No significant cell death was observed during 20 h of treatment time. Scale bar: 100μm.

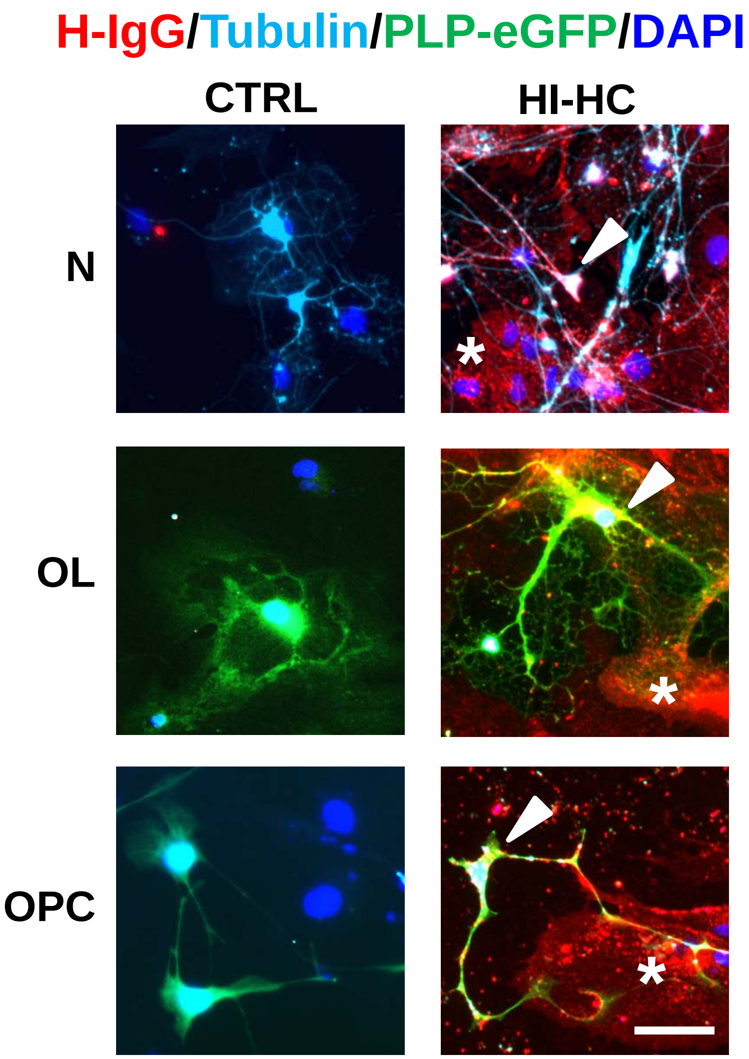

Figure S5: Binding of HC-derived human IgG to the surface of murine neurons, OLs, OPCs and astrocytes. Neuro-glial mixed cultures prepared from PLP-eGFP mouse pups were incubated with medium only (CTRL) or 5% heat inactivated HC (HI-HC) for 1 h. Cells were then fixed and stained for the neuronal marker β-Tubulin (Tubulin) and human IgG (H-IgG). H-IgG was detected on neurons, OLs, OPCs (arrowheads) and surrounding astrocytes (asterisk). Scale bar: 50µm.

Cytotoxicity (mixed culture)	N	OL	OPC	AST	AST (+NMO#53)
HC (*Complement Technology*)	yes	yes	no	no	yes
HC (*Sigma*)	yes	yes	no	no	yes
HC (*Innovative Research*)	yes	yes	no	no	yes
HC- single donor BM19 (*Innovative Research*)	yes	yes	no	no	yes
HC-single donor HM61 (*Innovative Research*)	yes	yes	no	no	yes
MC (*Complement Technology*)	no	no	no	no	no
MC (*Innovative Research*)	no	no	no	no	no

Table S1: Cytotoxic effects of multiple sources of human (HC) and mouse complement (MC) in neuro-glial mixed cultures. The sensitivity of neurons, astrocytes, mature oligodendrocytes, and oligodendrocyte precursor cells to HC and MC in the absence or presence of AQP4-specific rAb (+NMO#53). Human complement preparations pooled from multiple donors were obtained from Complement Technology, Sigma and Innovative Research. Single donor human complement preparations (BM19) and (HM61) were obtained from Innovative Research. Mouse complement preparations pooled from multiple animals were obtained from Complement Technology and Innovative Research. *Abbreviations: AST: astrocyte; N: neuron; OL: mature oligodendrocyte; OPC: oligodendrocyte precursor cell; AQP4, aquaporin-4.*
